# Supplementary material for: Impaired semantic control in the logopenic variant of primary progressive aphasia
Source: Brain Commun. 2024 Dec 21;7(1):fcae463. doi: 10.1093/braincomms/fcae463 (PMC11724431; doi:10.1093/braincomms/fcae463)
Supplement: fcae463_Supplementary_Data [file fcae463_supplementary_data.pdf]

Supplementary material

Supplementary Table 1 *Post hoc* tests for Bayesian ANOVAs across semantic tasks

Task	Groups		Prior Odds	Posterior Odds	$BF_{10,U}$	error %
Boston naming test	Control	tAD	0.414	10397.104	25100.829	$3.174x10^{-7}$
		lvPPA	0.414	807223.658	$1.2949x10^{+8}$	$1.525x10^{-9}$
		lvPPA+	0.414	$9.275x10^{+7}$	$2.239x10^{+8}$	$3.339x10^{-10}$
	tAD	lvPPA	0.414	70.365	169.876	$1.225x10^{-5}$
		lvPPA+	0.414	11080.967	26751.822	$4.390x10^{-8}$
	lvPPA	lvPPA+	0.414	2.151	5.194	$7.215x10^{-5}$
Cambridge Semantic Battery (CSB) naming	Control	tAD	0.414	1.765	4.262	$1.317x10^{-4}$
		lvPPA	0.414	58.001	140.026	$1.426x10^{-5}$
		lvPPA+	0.414	5620.969	13570.220	$9.795x10^{-7}$
	tAD	lvPPA	0.414	5.261	12.702	$5.870x10^{-5}$
		lvPPA+	0.414	252.768	610.237	$7.815x10^{-6}$
	lvPPA	lvPPA+	0.414	3.881	9.371	$5.310x10^{-5}$
CSB word-picture naming	Control	tAD	0.414	0.432	1.043	0.003
		lvPPA	0.414	1.274	3.075	0.009
		lvPPA+	0.414	4.640	11.203	$3.706x10^{-5}$
	tAD	lvPPA	0.414	0.347	0.839	0.003
		lvPPA+	0.414	1.205	2.908	0.007
	lvPPA	lvPPA+	0.414	0.432	1.043	0.003
Camel and Cactus Test	Control	tAD	0.414	4.125	9.959	$1.343x10^{-5}$
		lvPPA	0.414	38.315	92.501	$5.373x10^{-6}$
		lvPPA+	0.414	217.002	523.889	$4.932x10^{-6}$
	tAD	lvPPA	0.414	0.323	0.779	0.002
		lvPPA+	0.414	2.780	6.712	$3.086x10^{-5}$
	lvPPA	lvPPA+	0.414	0.677	1.635	0.005
Synonym judgement	Control	tAD	0.414	2.003	4.835	$1.003x10^{-4}$
		lvPPA	0.414	44.073	106.402	$1.770x10^{-5}$
		lvPPA+	0.414	8.381	20.235	$1.938x10^{-5}$
	tAD	lvPPA	0.414	0.321	0.776	0.002
		lvPPA+	0.414	0.994	2.399	0.005
	lvPPA	lvPPA+	0.414	0.527	1.273	0.003
Alternative object use task	Control	tAD	0.414	0.163	0.394	0.001
		lvPPA	0.414	23.539	56.828	$4.764x10^{-5}$
		lvPPA+	0.414	2.514	6.070	$1.907x10^{-4}$
	tAD	lvPPA	0.414	8.860	21.389	$1.574x10^{-5}$
		lvPPA+	0.414	1.314	3.172	0.006
	lvPPA	lvPPA+	0.414	0.241	0.581	0.001

Note: The first and second columns indicate the task and the groups being compared. The third and fourth columns indicate the adjusted prior model odds and the posterior model odds respectively. The posterior odds have been corrected for multiple testing by fixing to 0.5 the prior probability that the null hypothesis holds across all comparisons. Individual comparisons are based on the default t-test with a Cauchy (0, r=1/sqrt(2)) prior. As shown in the fifth column, the "U" in the Bayes factor denotes that it is uncorrected. The final column shows the numerical error of the Bayes factor computation.

Supplementary Table 2 Loadings for principal component analysis of executive tasks

**Measure**	**PC 1 (“Executive” scores)**
Trail Making Test B	**0.92**
Digit Span Backward	**0.58**
Brixton	**0.81**
Raven’s Coloured Progressive Matrices	**0.79**

Rotation: Orthogonal varimax. Loadings above a threshold of 0.5 are bolded.

Supplementary Table 3 Voxel-based morphometry results showing group differences in grey and white matter intensity. Voxel-wise differences of grey and white matter intensity between each of the patient versus control groups were assessed using independent *t*-tests, with age and total intracranial volume included as nuisance variables. Clusters were extracted, corrected for family-wise error at $P < 0.05$, with a cluster threshold of 100 contiguous voxels.

Comparison	Regions	Hemisphere	Number of Voxels	Peak MNI coordinates			t-value
All patients < controls	Temporal lobe including lateral and medial regions and the insula	Left	149799	-50	-34	0	12.04
	Middle frontal gyrus	Right	503	44	12	32	5.70
	Parietal lobe including the angular gyrus and inferior parietal regions	Right	473	39	-60	40	5.84
	Inferior parietal lobule	Right	166	51	-44	45	5.46
	Postcentral gyrus	Right	130	39	-28	45	5.18
	Anterior superior temporal gyrus	Right	116	32	20	-28	5.81
tAD < controls	Medial and lateral temporal including the hippocampus, fusiform and middle temporal gyri	Left	23323	-34	-16	-16	7.60
	Medial and lateral temporal including the parahippocampal, fusiform and middle temporal gyri	Right	11845	32	-24	-18	7.35
	Superior medial frontal	Left	204	-8	36	40	5.90
	Medial frontal	Right	103	8	32	-16	5.36
	Insula	Right	131	42	0	6	5.66
lvPPA < controls	Temporal lobe including medial and lateral regions, particularly the middle temporal gyrus, extending posteriorly into the parietal and occipital regions	Left	31376	-66	-21	-8	8.83
	Inferior temporal lobe including the fusiform gyrus	Right	567	57	-20	-27	6.45
	Middle temporal gyrus	Right	282	66	-12	-10	6.10
	Inferior temporal gyrus	Right	137	58	-38	-20	5.55
	Medial frontal gyrus	Left	112	-6	40	33	5.75
lvPPA+ < controls	Temporal lobe extending subcortically and medially, including hippocampus, anteriorly, and posteriorly into the parietal and temporo-occipital lobes	Left	31212	-42	-22	-18	8.92
	Medial temporal, including hippocampus, fusiform, and parahippocampal gyri	Right	2019	39	-20	-22	6.48
	Inferior parietal lobule including the supramarginal gyrus	Left	769	-50	-44	48	6.59
	Middle frontal gyrus	Left	257	-46	27	26	5.56
	Parietal lobe	Left	204	-27	-57	42	5.76
	Middle temporal gyrus	Right	107	56	-30	-3	5.52

lvPPA, logopenic variant of primary progressive aphasia; tAD, typical Alzheimer's disease.
